# The impacts of COVID-19-induced online lectures on the teaching and learning process: An inquiring study of junior secondary schools in Orlu, Nigeria

**DOI:** 10.3389/fpubh.2022.1054536

**Published:** 2022-11-21

**Authors:** Confidence Chioma Nneji, R. Urenyere, Kingsley Eghonghon Ukhurebor, Saheed Ajibola, Olatunji Oluwatosin Onaseso

**Affiliations:** ^1^Africa Centre of Excellence on Technology Enhanced Learning (ACETEL), National Open University of Nigeria, Abuja, Nigeria; ^2^Department of Physics, Faculty of Science, Edo State University, Uzairue, Edo State, Nigeria; ^3^SAGE Group Technologies, Hazlet, NJ, United States

**Keywords:** COVID-19, learning, online lecture, student, teaching

## Abstract

This study investigated how the sudden shift in the system of learning during the COVID-19 pandemic impacted the students, how the external environment impacted their performance, and the structural barriers encountered, which equally had significant impacts on students at junior secondary schools (JSS) in Orlu, Imo State, Nigeria. The study adopted the descriptive survey research method. The simple random sampling method was adopted with a sample size of 650 students. The data were collected using a structured questionnaire, rated using a four-point Likert scale, and analyzed using descriptive statistics such as frequency counts, percentages, and means. 60.10, 58.80, 59.50, 59.00, and 59.50% of the respondents agreed to research questions respectively. Based on these results, it was concluded that the COVID-19-induced online teaching and learning impacted negatively on the students and on the process of teaching and learning due to inadequate prior preparation for such a system of teaching and learning at the JSS level within the study area. These have serious implications and remain significant for policy and practice in the education sector.

## Introduction

Nigeria is known for many lofty policies, but with little effort put into their sincere implementation. There is no denying the fact that Nigeria loves a fire-brigade approach to issues (1). This position was attested to and played out during the emergence of the global pandemic known as the COVID-19 pandemic (2–4). This pandemic impacted all sectors of global society (5, 6), with educational institutions bearing the brunt of the damage (2, 3). Its impacts on the education sector remain highly felt and of utmost concern (2–4). This is because the growth and development of every society or nation depend a lot on the progress of its education sector (7). Education, according to Whitehead (8), remains the bootstraps through which nations and civilizations lift themselves up. Also, the future of any nation or state depends a lot on the quality of education received by its youth, whose education has been challenged, if not threatened, by the pandemic (8). The COVID-19 pandemic brought many upheavals in different sectors (5, 9, 10). In order to take care of the spread of the pandemic, there was a global lockdown, which adversely affected the school programs as they were under lock and key (2, 3). When it dawned on many societies and governments that the solution to the pandemic is far-fetched and that an endless wait is not an ideal decision, there was a resort to online lessons.

Many nations had earlier adopted the policy of online education, but Nigeria's remained only on the policy document. No single conventional university can fully brag about complete online education. As other countries resorted to online lessons, which were already a part of their education programs and systems, Nigeria jumped on board with no feasibility studies or adequate infrastructure in place. This was a herculean challenge to many higher institutions. With such a scenario at the tertiary levels of education, one wonders what will be obtainable at the secondary school levels. Generally, in the Nigerian situation, there was no emergency system on the ground for such a situation. There were no adequate human and material resources to face such a challenge. Many teachers lack basic computer and internet skills. The same goes with the students. These are what were available on the ground when the government came up with the executive fiat that schools should resume lectures and lessons online. With such an uncoordinated and unprepared situation at the tertiary level, one can then imagine and have a picture of what would be obtainable at the secondary school level. The nations that were already into online lectures started with it on a stage-by-stage basis (11, 12). This gave students options that allowed them to take into consideration their different learning abilities. This was not the case in Nigeria. Nigeria woke up overnight and jumped into online lectures without adequate preparations. This attests to Nigeria's position of taking a fire brigade approach to issues. With this situation, one can predict what the experience would be for secondary school students. With such a development, students were forced to abruptly jump from the traditional face-to-face method to an online, virtual method devoid of any physical interaction with both teachers and students. This has various social and psychological implications and effects on these secondary-school students (13, 14). At their age, secondary school students are still protected from the open society and its enemies (15). Many students need their teachers to stand in loco parentis. With such a policy and abrupt changes in learning methods (online lessons), many variables come into play and affect the students in many ways. Such variables range from the environment to the social, psychological, financial, and other factors obtainable in both the individual and Nigerian settings. Some have caused serious confusion and miseducation among these students.

The “United Nations Educational, Scientific and Cultural Organization (UNESCO)” (16), outlined certain recommendations for nations wishing to embark on online lectures for the sake of COVID-19. One is to examine the readiness and choose the most relevant tools, bearing in mind high technology and low technology solutions based on the reliability of local power supplies, internet connectivity, and the digital skills of teachers and students. This paper addresses one issue: assessing Nigeria's readiness for these novel and emergency online lectures, particularly at the JSS levels. Also, UNESCO (16), insisted that there must be measures to ensure that students, including those with disabilities or from low-income backgrounds, have access to distance learning programs. Part of the quest of this study is to assess the impacts of this emergency study, or teaching and learning, on the economic and social backgrounds of the students, which affects their capacities for adapting to the demands of the online system. From both the Nigerian setting and the UNESCO recommendations, the COVID-19-induced online lectures involved lots of narrative and consequences for the process of teaching and learning. This is the challenge of this study, to unveil such a successful pedagogy.

If instructional materials can constitute distractions in the traditional face-to-face classroom based on their introduction and removal (timely), one can then imagine what would happen when the only instructional material is the smartphone held by the students (17–21). This has no timely introduction or removal. It has to be noted that the majority of these secondary school students are just encountering these devices for the first time. This is a critical factor in this research study. According to scholars like Pestalozzi (22) and Rousseau (23), the education of the child must match his or her stages of development, levels of awareness of the environment, and cultural facts. This plays an enormous role in the successful teaching and learning of the children (17). These are matters of concern in a Nigerian setting where many of the online technologies and tools remain alien to many JSS students (24). Another factor or problem is that sometimes parents are not around to regulate the strict use of these devices needed for the online lectures. Equally, many of the teachers and students do not have the basic skills needed for this novel online method of teaching and learning. This has many consequences for the students (24). This broadly constitutes the fulcrum and major focus of the problems addressed in this research study. In view of the above, the following becomes problematic and draws the attention of this study:

- Nigeria is still struggling to switch from an analog to digital society in this millennium. This is still a pipe dream, as many sectors and subsectors of Nigerian society still operate on an analog basis. With such a situation on the ground, the problem becomes manifest: whether Nigeria is prepared for the sudden decision and policy to adopt the online system of education, especially at the secondary school level. This is keeping in mind that previous efforts to operate and implement such a policy at the tertiary levels have not yielded any success.
- Another concern and problem emanating from the situation and scenario that needs serious investigation is whether these secondary school students were given prior and adequate orientation to this new mode and process of teaching and learning.
- The orientation of secondary school students in view of the new online system of teaching and learning involves extensive narratives about the socio-economic consequences surrounding it. These include the provision of smartphones, laptops, desktops, Internet, Wi-Fi, and other online media technologies. The online narrative involves the parents' economic and academic backgrounds. Thus, the problem here once more, is whether all these parties are well-disposed and motivated toward this new method of teaching and learning at the secondary school levels. A lot of variables are involved here which range from structure to the, disposition of students(readiness) to) economic, political, social, cultural, administrative factors, etc.
- The environment remains strategic in the process of teaching and learning, with special attention to children, especially at the secondary school levels. From the social-epistemological perspective, learning is socially constructed. How does a sudden shift from a social environment of learning, like a classroom, to a virtual and individualized, if not isolated, environment affect the interest and concentration of the students? Students learn faster and are easily motivated by association and the presence of a peer. How does the absence impact these students in an isolated manner? Many parents and guardians are intellectually incapacitated to assist their wards at home; many do not have a phone, much less understand the operations of technology. Some may be as frustrated as the students. This is a problem and challenge to online education that needs to be assessed properly.

There is no gainsaying the fact that not all teachers can manipulate and adapt to the use of online media technology for teaching and learning. Many are not literate computer and internet wise. In such a situation, how can learning be effectively pursed in such a situation? Wouldn't there be a communication/knowledge gap in the achievement of the objectives of the lessons? Are not the students at the losing end? These constitute the major problems that remain the focus of this study. Simply put, these are:

- Is Nigeria prepared for the novel online system of education at the secondary school level?
- Were students given prior and adequate orientation to the new mode of teaching and learning?
- Would the sudden change in the teaching and learning environment not have certain negative effects on the students (in the absence of adaptive mechanisms)?
- From the perspective of teachers who are not fully computer and internet savvy, the achievement of the objectives of the lesson may not be realized, thus having negative impacts on the wealth of knowledge expected of the students.

Hence, the purpose of the study is to access the impacts of the COVID-19-induced online lectures on the process of teaching and learning in junior secondary schools (JSS) as seen from the perspective of Orlu, Imo State, Nigeria. Successful pedagogy involves a lot of planning and execution and not a haphazard approach and policy. To achieve the purpose, the following constituted research questions were used to determine the investigative focus of the study:

- Research Question 1: What are the impacts of the sudden shift from traditional face-to-face learning to online learning on JSS students?
- Research Question 2: Did the external environment affect the performance of the students compared to the traditional face-to-face learning system?
- Research Question 3: Were there structural barriers encountered that hampered their successful participation in the online lectures?
- Research Question 4: Are the academic performances (APs) truly a result of the effects of the students in a non-regulated environment, or were there exogenous influences from third parties?
- Research Question 5: Did all the teachers have the basic skills for successful delivery of online lectures?

This is why this study set out to achieve the following purposes:

- To find out the impacts the COVID-19-induced online lectures had on the process of teaching and learning for students.
- Assess the impacts of a sudden shift from traditional face-to-face teaching and learning to the virtual/online system of teaching and learning.
- To assess Nigeria's and students' readiness for a successful shift and use (adoption) of the online education system at the JSS level.
- To find out if the lack of basic computer skills of the teachers hampered the online lesson delivery.
- To find out if there are structural barriers to student's online lessons performances.

Agreeably, COVID-19 was an unexpected pandemic around the globe. The lockdowns impacted almost all societies and countries. However, the emergency steps taken by the Nigerian government to minimize the damage, particularly in the education of the students, are commendable but not sufficient and adequate. Nonetheless, the purpose of this research is to uncover, assess, and critically analyse the effects of such a sudden and uncoordinated shift from traditional face-to-face teaching and learning to virtual teaching and learning on JSS students. The abrupt and uncoordinated shift must have had its impact on parents, especially those from economically backward backgrounds. Allegedly, this will definitely negatively affect the students.

## Methods

This section deals with the methods, processes, and procedures adopted for the study, which are organized under the following sub-headings: research design, population, sample and sampling technique, instrument for data collection, validation and reliability of the study, method of data collection and analysis.

### Research design

For the achievement of the objectives of the study, a survey design was used to provide and collect data from members of the population with respect to one or more variables. It permits description in a natural setting. Data collected are described in a systematic manner, revealing the characteristics or facts about a specified population. The work seeks to determine and improve the current behavior exhibited by a particular population in different settings such as schools, communities, and the workplace.

### Population, sample and sampling technique

Orlu is one of the biggest towns in Imo State, Nigeria. It is about 39 km south of Owerri, the capital of Imo State. It has the following boundaries: on the north, Ideato; on the south, Nkwerre; on the east, Isu; and on the west, Orsu and Ihiala of Anambra State. Orlu covers approximately 202 km^2^ with a population of almost 220,000. They are settled in well-defined groups called autonomous communities such as Amaifeke Umuna, Owerre-Ebeiri, Ihioma, Umuowa, Okporo, etc. (25, 26).

As of June 2022 (when this study commences), there are 53 secondary schools in the area with a total of about 21,569 students. The target population was 2,212 students consisting of JSS students in Urban Secondary School, Community Secondary School, Girls Secondary School, Ihioma, St. Joseph Secondary School, and Umuna in Orlu, Imo State, Nigeria. These schools are the most prominent ones that are run by the government, except for St. Joseph Secondary School, Orlu, which is run by the Catholic Church. However, the availability of computers, uninterrupted internet, and teacher/student ratio are somehow not adequate but identical and are better than other schools within the study area. The sample size for this study was 650 respondents randomly selected from JSS students in five (5) out of fifty-three secondary schools in Orlu. JSS students from each of the five secondary schools were chosen using random sampling (both male and female).

### Instrument for data collection

A developed structured questionnaire was used. Section Introduction of the questionnaire entailed the respondents' demographic data. Section Methods of the questionnaire elicited information that answered research questions as stated in section Introduction of this study. The scale consisted of 20 items rated on four-point Likert scale coded as follows: “Strongly Agree (SA), Agree (A), Disagree (D), Strongly Disagree (SD)”.

### Validity and reliability of the instrument

The instrument was validated by experts in measurement and evaluation as well as experts in the sociology of education. These experts scrutinized the work along with the objectives of the study and research questions in order to ascertain the clarity, relevance, adequacy, and possibility of the instrument in eliciting appropriate responses for the study. Modifications were made by the researcher based on the comments and suggestions of the experts.

A test-retest statistic method was utilized to test the reliability of the instrument, using Pearson product moment correlation. The correlation coefficient was 0.84, showing that the instrument was reliable and good for the study.

### Data collection and analysis

Research assistants were trained to help in the administration of the instrument and were distributed to respondents. The distribution was done on a face-to-face basis. On-the-spot collection ensured a high percentage of the completed questionnaire would be returned. The duly completed copies of the structured questionnaire schedule were collected, coded, and analyzed using descriptive statistics of frequency counts, percentages, and mean to answer the research questions.

## Results and discussions

The data collected were analyzed using descriptive statistics of frequency, such as percentage and mean, to answer the research questions.

### Research question 1

What are the impacts of the sudden shift from traditional face-to-face learning to online learning on JSS students?

According to Table 1, 60.10% of the sample students strongly believed that the abrupt transition from face-to-face learning to online learning had a significant impact on teaching and learning. 30.03% agreed, whereas 5.50% disagreed, and 3.50% only disagreed strongly on the impacts of the online learning method. Because the majority of respondents strongly agreed, the researcher strongly agreed that the sudden shift to online teaching and learning by JSS students has significant implications for their teaching and learning process. Also, the mean score in all the four items exceeded the mean cut-off point of 2.50. The grand mean of 3.80 indicated that the general decision rule is accepted. It is therefore concluded that the sudden shift to online learning has had a great impact on the process of teaching and learning among the JSS students, as seen in the responses, and this is in conformity with the results from regions (27–29).

**Table 1:** Responses on the impact of a sudden shift to online learning on JSS students.

**No**	**Item**	**SA**	**A**	**D**	**SD**	**Total**	**Mean**
		***F*** **/%**	***F*** **/%**	***F*** **/%**	***F*** **/%**	***F*** **/%**	***X***
1.	Online learning is new to JSS students, and it involves devices like smart phones, the internet, and laptops, which they have less knowledge of or access to.	350/54.30	250 /38.50	30/4.60	20/3.10	650/100	3.80
2.	JSS students require proper and adequate orientation to this new mode of teaching and learning.	420/64.60	180/27.70	35/5.40	15/2.30	650/100	3.70
3.	Online learning involves a lot of variables involving students' readiness, structure, economics, socialization, culture, and administration to make it work effectively.	390/60.00	210/32.30	30/4.60	20/3.10	650/100	3.80
4.	Online learning is individualized; interest and concentration are reduced because they are not with the teacher and peers.	360/55.30	240/36.90	35/5.40	15/2.30	650/100	3.80
5.	Cooperation with peers and teachers boosts motivation more than being alone with unfamiliar gadgets.	400/61.50	150/23.10	55/8.50	45/6.90	650/100	4.00
6.	Some parents are incapacitated both intellectually and financially to support their wards' online learning at home.	420/64.60	180/27.70	30/4.60	20/3.10	650/100	3.60
	Total	23/40.00	12/10.00	21/ 5.00	13/50.00		
	Average (%)	60.10	30.03	5.50	3.50		
	Grand mean						3.80

### Research question 2

Did the external environment affect the performance of the students compared to the traditional face-to-face learning system?

From Table 2, it can be seen that the respondents' percentage who strongly agreed that the external environment has influence over the performance of students as compared to traditional face-to-face learning were 58.80%. 32.02% agreed, whereas 5.94% disagreed and only 3.30% strongly disagreed on the environmental influences. Since the majority of the respondents strongly agreed that the enlisted affected their performance in an external environment, the researcher also believed that these external factors, affected the performance of the students academically as far as online learning in some homes. The results of the mean score of all the respondents and the grand mean of 3.74 indicated that the decision rule is accepted. The conclusion was therefore made that these external factors adversely affected the AP of JSS students as deducted in schools in Orlu, Imo State, Nigeria, and this is in conformity with the results from regions (11, 12, 27–29).

**Table 2:** Responses to the impacts of the external environment on APs compared to traditional face-to-face learning.

**No**	**Item**	**SA**	**A**	**D**	**SD**	**Total**	**Mean**
		***F*** **/%**	***F*** **/%**	***F*** **/%**	***F*** **/%**	***F*** **/%**	***X***
7.	There are so many distractions at home, like house chores, that may hinder good performance by the students.	420/64.61	150/23.08	50/7.70	30/4.60	650/100	4.10
8.	Smart phones, which students use, may bring other things into their minds like watching immoral videos, playing games, etc., especially when they are not properly guided.	380/58.50	200/30.80	50/7.70	20/3.10	650/100	3.80
9.	Parents and guardians are not always around or available to provide the devices needed for them to solve their tests and assignments online, and they will perform poorly.	350/53.80	250/38.50	30/4.60	20/3.10	650/100	3.50
10.	Some parents are illiterate, and students may not have knowledge of the use of smart phones; they become incapacitated and do not participate in the online lessons.	400/61.53	200/30.80	28/4.31	22/3.40	650/100	3.60
11.	Parents who are buoyant financially will not be able to provide the laptops, phones, or even buy data to make online learning a success, and so students perform poorly.	360/55.38	240/36.92	35/5.40	15/2.31	650/100	3.70
	Total	1,910	1,040	193	107		
	Average (%)	58.80	32.02	5.94	3.30		
	Grand Mean						3.74

### Research question 3

Were there structural barriers encountered that hampered their successful participation in the online lectures?

From Table 3, it shows that 59.50% of the respondents strongly agreed that there are barriers encountered by the students that hampered their participation in online lectures, such as a lack of steady electricity, poor networks due to the area of residence (remote), a lack of devices to use for online lectures like smartphones, etc. While, 32.85% agreed also to these and about 5.13% disagreed and only 2.60% of the respondents strongly disagreed. The researchers strongly believed that these barriers hampered the participation of most students among the majority who responded. Also, using the grand mean of 3.70, it is generally accepted and concluded that these barriers hampered the participation of some students in online lectures, and this is in conformity with the results from regions (13, 15, 30–32).

**Table 3:** Responses on the structural barriers that hampered the students' successful participation in the online lectures.

**No**	**Item**	**SA**	**A**	**D**	**SD**	**Total**	**Mean**
		***F*** **/%**	***F*** **/%**	***F*** **/%**	***F*** **/%**	***F*** **/%**	***X***
12.	Some students live in a remote area where networks are not available and there is an unsteady electricity supply to charge phone or laptop batteries. These are the barriers to their participation in online lectures.	360/55.3	240/36.90	30/4.60	20/3.10	650/100	3.60
13.	Some students' parents cannot afford to buy them Android phones or other devices to use for online classes.	420/64.61	180/27.7	40/6.20	10/1.53	650/100	3.80
14.	Some have no prior knowledge of the use of the devices and cannot pay for a business center, so the child's participation is hindered.	380/58.50	220/33.80	30/4.60	20/3.10	650/100	3.70
	Total	1,160	640	100	50		
	Average (%)	59.50	32.80	5.13	2.60		
	Grand mean						3.70

### Research question 4

Are the APs truly a result of the effects of the students in a non-regulated environment, or were there exogenous influences from third parties?

From Table 4, it can be deduced that a majority of the respondents strongly agreed that the AP of the students also comes from exogenous influences, as shown by 59.00% strongly agreeing to the fact that people other than the students themselves can do online exams, tests, etc. for them and some cannot cope because they are all alone, 33.3% also agreed, whereas 5.40% disagree strongly, and only 2.31% disagree strongly to this. It is believed by the majority that exogenous influences such as these could affect the students' performance and not truly be a result of an unregulated environment alone. The mean score of the four responses yielded a grand mean of 3.80, which can be generally used to accept the decision rule. It is therefore concluded that the AP of the students, sometimes good or poor, is not truly a result of the non-regulated environment alone but also as a result of exogenous influences such as lack of communication with teachers, not being conversant with the devices, and examinations being written by other people other than the students themselves, as seen in this study and this is in conformity with the results from regions (27–29).

**Table 4:** Responses to the exogenous influences on the AP of the students, which are not only a result of a non-regulated environment.

**No**	**Item**	**SA**	**A**	**D**	**SD**	**Total**	**Mean**
		**Fe/%**	***F*** **/%**	***F*** **/%**	***F*** **/%**	***F*** **/%**	***X***
15.	Some students may not do their assignments or tests on their own, but parents and others do it for them, and they perform well, which is a disadvantage to the student.	420/64.61	180/27.70	40/6.20	10/1.53	650/100	4.00
16.	Students who are good with instructional materials may not do well with reading or doing exams timed online with a laptop or smartphone.	350/53.80	250/38.50	30/4.60	20/3.10	650/100	3.60
17.	Students who stay with older parents may not know who to channel the questions they find difficult to when their teachers and peers are not around to communicate and answer them. This hampers their performance.	380/58.50	220/33.80	35/5.40	15/2.31	650/100	3.81
	Total	1,150	650	105	45		
	Average (%)	59	33.33	5.40	2.31		
	Grand mean						3.80

### Research question 5

Did all the teachers have the basic skills for successful delivery of online lectures?

From Table 5, it shows that 59.50% of the respondents strongly agreed that not all the teachers have the basic skills for successful delivery of online lectures; 31.30% agreed also, whereas 5.90% disagreed and only 3.33% strongly disagreed. The grand mean of 3.8 also shows the decision rule, and so the researcher guessed and believed by the majority that some teachers lack basic skills, while some are computer illiterate, some are not really conversant with delivering lectures online, and some cannot evaluate the students at all. Their responses are ascertained from JSS in Orlu, Imo State, Nigeria, and this is in conformity with the results from regions (17, 30, 31, 33).

**Table 5:** Responses as to whether all the teachers have the basic skills for successful delivery of online lectures.

**No**	**Item**	**SA**	**A**	**D**	**SD**	**Total**	**Mean**
		***F*** **/%**	***F*** **/%**	***F*** **/%**	***F*** **/%**	***F*** **/%**	***X***
18.	Most of the teachers are not computer-literate and need proper training for this online learning.	380/58.50	220/33.80	35/5.40	15/2.30	650/100	3.80
19.	The lesson objectives may not be achieved or delivered as a result of a lack of basic skills for online learning by some teachers, and this may have a negative impact on the expected knowledge or performance of the students.	420/64.61	150/23.08	50/7.70	30/4.60	650/100	4.00
20.	Teachers who are computer literate will not have face-to-face interaction to evaluate students or discuss with them via questions to determine whether or not they understood the lectures.	360/55.30	240/36.90	30/4.60	20/3.10	650/100	3.50
	Total	1,160	610	115	65		
	Average (%)	59.50	31.30	5.90	3.33		
	Grand mean						3.80

Even though the ranks of these selected students in their previous class were not mentioned directly in evaluating their APs. However, it is obvious from the analysis of these research questions that their AP is consistently better during direct contact of student-teacher classes. Hence, from the data presented and analyzed, the following are the summarized results.

Online learning and lectures have great impacts on the students of the JSS. They had to suddenly shift to this online system from face-to-face interaction, which they are used to. They normally use instructional materials like books and chalkboards; they visualize teachers and peers and interact, and then suddenly shift to a learning method that uses devices, i.e., laptops and android phones, which use networks and electricity, which they have not mastered, and thus the readiness to learn is not there, and this is in conformity with the results from other regions (27–29).

When compared to the traditional face-to-face learning system, students' AP was influenced by their external environment, such as those with illiterate parents who know little about smartphones, financially prosperous parents who cannot afford to buy or pay for online materials, and those who have no guardian available to guide them. Students deviate to videos and games; some only house chores distract them, and this is in conformity with the results from regions with similar terrain (30, 31).

Some students' successful participation in online learning was hampered by structural barriers, as they live in remote areas with no electricity, no networks, and even no smartphones or laptops to work with and this is in conformity with the results from regions with similar terrain (30, 31).

The students' AP is not truly a result of the non-regulated environment alone, but rather of exogenous influences, such as those who will not do the online tests and exams by themselves but by their parents, siblings, and so on, the knowledge is not there. The students are at a disadvantage and this is in conformity with the results from regions (27–29).

Not all the teachers have the basic skills for successful delivery of online lectures, as some of them are not trained for this and are computer illiterate, and this is in conformity with the results from regions with similar terrain (30, 31).

## Conclusion, implications, limitations, and recommendations

In summary, this study has a purpose, a significance, literature to review, data to analyse, and the disclosure of specific findings that establish the study's point of view. The purpose of this study was to assess the impact of the COVID-19-induced online lectures on the process of teaching and learning. A descriptive survey research design was adopted for the total population of 650 students, with a sample size drawn using simple random sampling. Five schools out of 53 secondary schools in the area (Orlu, Imo State, Nigeria) with a population of 1,212 students in JSS were used. A personally structured, valid, and reliable questionnaire was used to collect information on the study. The data collected was analyzed using percentages, frequency, and mean to answer the research question.

The discussion of findings was done in regards to the five research questions. It was revealed that there are influences or impacts from the sudden shift from classroom learning to online learning for the JSS students. There are so many distractions from house chores and diversion to other sites while using smartphones; parents and guardians are not always available to provide the devices needed for their homework, and they perform poorly; the cost of payment for online materials, which some parents cannot afford; illiteracy or not being computer literate; and lack of prior knowledge of the use of the gadgets all contributed to influence their performance in online learning. As it was are seen from the result, that not having internet facilities at residential area, shortage of electricity supply, cost of purchase of smartphone and laptop or even pay for a business center and the child's participation is hindered. It was also revealed in the results of the analysis that some students actually do not carry out online assignments on their own and may score highly, which is a disadvantage to them because learning is supposed to be from previous experiences. Students who are good with instructional materials like books, however, may not perform well with examinations set and timed online. Some students may not know to whom to direct difficult questions because their teachers and classmates are not around them. Lastly, it was revealed that some teachers are not computer literate and need adequate training before they can successfully deliver online lectures.

The process of teaching and learning involves a lot, and every bit of knowledge must contribute to human development for it to be worthwhile. In view of this, the results of the study have some implications/consequences for students, parents, and teachers. Children learn from experience, and a sudden uncoordinated shift from the usual face-to-face or classroom system to an online system would not be easy for all at the start. An interesting and flexible online education should have some fundamental components that can make its operation smooth, accessible, and affordable. The system should be incorporated into the school from the very beginning and done under the supervision of both teachers and parents at home. The gadgets to work with should be provided, and they should not be done in a haphazard manner. The environment can make or break a child; more emphasis should be placed on theories of readiness to learn from the standpoint of factors exogenous to the learner and his disposition. The results revealed several factors that inhibit successful online lectures from social, economic, and cultural perspectives. Thus, this study will contribute to the quest to ensure successful teaching and learning by ensuring the policies necessary for such are well implemented. The study has great implications for validating the constructivists' positions on the relevance of the environment in successful pedagogy.

There can never be any work without some hurdles in the form of limitations. Hence, this study faced a few limitations as follows:

- The schools were repeatedly visited before permission was given to interact with the respondents.
- Some of the respondents were biased in their answers to the questions in the questionnaires.
- COVID-19 and the measures put up also hindered close interaction.

There appear to be several fundamental components to an engaging and adaptable online education that can make it run well, be available to everyone, and be reasonably priced. To enable complete embrace in the near future, some fundamental resources, such as the internet and electrical devices like computers, laptops, and smart phones, must be put in place. Hence, the following recommendations should be given appropriate consideration:

- Government and privately owned schools should make online resources accessible early enough for school students.
- Students should be exposed to the use of a computer as early as elementary schools such as kindergarten.
- Teachers should be trained and retrained on the use of computers so that their teaching can become more effective and students can be exposed to practical computer classes through them.
- The government should make the internet available and accessible.
- There should be free Wi-Fi so that students get used to online programs.
- Governments and school administrators should assist in grooming and monitoring their students during online classes to avoid divided attention and to help them manage their time.
- Further research should be conducted in areas such as: evaluating the cost-effectiveness of computer-assisted instruction; keeping up with online learning; using an internet-based technology to improve performance; evaluating evidence-based practices in online learning; factors influencing student academic achievement in virtual schools; the benefits and challenges of blended learning environments; and providing feedback opportunities in online learning.

## Data availability statement

The original contributions presented in the study are included in the article/supplementary material, further inquiries can be directed to the corresponding author/s.

## Author contributions

All authors listed have made a substantial, direct, and intellectual contribution to the work and approved it for publication.

## Conflict of interest

Author OO was employed by SAGE Group Technologies. The remaining authors declare that the research was conducted in the absence of any commercial or financial relationships that could be construed as a potential conflict of interest.

## Publisher's note

All claims expressed in this article are solely those of the authors and do not necessarily represent those of their affiliated organizations, or those of the publisher, the editors and the reviewers. Any product that may be evaluated in this article, or claim that may be made by its manufacturer, is not guaranteed or endorsed by the publisher.
